# Bibliometric Insights into Research on Frailty and Falls

**DOI:** 10.4314/ejhs.v34i4.9

**Published:** 2024-07

**Authors:** Norbayah Zainal, Azliyana Azizan

**Affiliations:** 1 Department of Healthcare Professional, Faculty of Health and Life Sciences, Management and Science University (MSU), University Drive, Off Persiaran Olahraga, 40100 Shah Alam, Selangor, Malaysia; 2 Centre of Physiotherapy, Faculty of Health Sciences, Universiti Teknologi MARA, Puncak Alam, Selangor, Malaysia

**Keywords:** Bibliometric analysis, Frailty, Fall, Older adults

## Abstract

**Background:**

Global population aging has sparked research into frailty and falls given their impact on older adults. This study provides a bibliometric analysis of frailty and fall literature to identify publication trends, leading contributors, impactful works, and conceptual themes.

**Methods:**

Frailty and fall publications were retrieved from Scopus and Web of Science databases without date restrictions. Data was analyzed using ScientoPy, and VOSviewer to generate statistics, visualizations, and maps based on temporality, productive countries, institutions, citations, subject categories, and keyword occurrences.

**Results:**

After pre-processing, 345 publications remained (84.6% Web of Science, 15.4% Scopus). The literature has grown steadily since 1990, led by the United States, China, and Japan. Prolific institutions were identified, including Pittsburgh University. Highly cited impactful studies were published across journals like the Journal of the American Geriatrics Society. Geriatrics/gerontology was the dominant subject category. Keyword co-occurrence analysis revealed clusters focusing on geriatric physical health, cardiovascular health, cognition, interventions, and mortality.

**Conclusions:**

This bibliometric analysis synthesizes a comprehensive overview of frailty and fall research, identifying rising publication and citation trends, leading global contributors, impactful studies, and thematic focuses. The findings can inform resource allocation, international collaboration, impactful evidence utilization, and future research planning to advance frailty science and clinical care for older populations. Ongoing investigation is warranted into frailty mechanisms, assessment, management, and multidomain interventions.

## Introduction

Global population aging is expected to increase dramatically, from 461 million adults over 65 years old in 2004, to an anticipated 2 billion by 2050 (1,2). The clinical state of frailty is the most troublesome manifestation of population aging (3). Frailty is a complex health state associated with aging that is characterized by weakness, decreased physiologic reserve, and increased vulnerability to stressors (4). The concept has evolved over the past few decades from simple definitions based on dependence and debility to more nuanced multidimensional models encompassing medical, psychological, and social factors. Two main approaches have emerged for measuring frailty, the frailty index which defines it as a nonspecific risk state marked by sarcopenia, weight loss, and exhaustion; and the frailty phenotype which views frailty as a distinct clinical syndrome marked by the same three key criteria (5). Frailty and falls are reciprocity linked, and understanding this connection is paramount in the field of geriatrics and healthcare for older adults. While numerous research has shown that frail older adults have higher risk of falls (6-9), other studies have also consistently reported a higher risk of falls among frail older adults associated with other disabilities including visual impairment compared to their non-frail counterparts (10-13).

The spectrum of frailty is broad and covers all aspects including physical, psychological, cognitive, and social development. Physical impairment is inclusive of muscle weakness, gait instability, and balance deficits, thus increasing vulnerability to falls and leading to mobility decline and higher mortality rates among older adults (14). A cross-sectional study by Niksolat et al., in 2022 found that older adults with rheumatoid arthritis has more features of geriatric syndrome compared to younger adults, and eventually are more prone to have a cognitive impairment, fall and functional disability (15).

Normal aging brought about changes in cognition, including reductions in working memory, executive cognitive function, and processing speed. These age-related brain changes are due to alterations in neuronal structure without neuronal death, loss of synapses, and dysfunction of neuronal networks (16). Older adults with both physical frailty and cognitive impairment are shown to be at higher risk of fall falls as a result of sensorimotor integration impairment which also leads to adverse health outcomes, including death, disability, hospitalization and incident dementia, than those with either condition alone (17).

Psychological frailty experienced older adults incorporate various symptoms including anxiety, depression and low mood that led to heightened physiological emotions, disrupted concentration, and a decline in desire for physical exercise, all of which can impair equilibrium and raise the risk of falls (18,19). Older adults with depression also perceived themselves as unhealthy, which also contributed to fall (20). Recognizing the importance of this issue, several systematic reviews and metaanalyses have been conducted to synthesize the existing literature on frailty and falls. A recent systematic review by de Souza et al. (2022) examined the association between frailty and falls, including 10 articles with a total of 6 294 participants. The review found that frail older adults had a significantly higher risk of falls compared to non-frail individuals, with a pooled odds ratio ranging from 1.04 (95% CI = 1.02; 1.07) to 7.16 (95% CI = 2.34; 21.89) (21). Another meta-analysis by Yang et al. (2023) focused on the relationship between different frailty components and falls, highlighting the importance of other factors such as gender, areas, level of national economic development, and healthcare manager's understanding of frailty (22).

While these reviews provide valuable insights, there is a need for a comprehensive bibliometric analysis to examine the overall landscape of research on frailty and falls among older adults. Bibliometric studies can uncover publication trends, identify leading contributors, and reveal conceptual themes and research gaps (23,24). Such analyses can inform future research directions, resource allocation, and international collaborations in this critical area.

The present study aimed to conduct a bibliometric analysis of frailty and falls among older adults. Four key objectives were addressed:

1. What has been the quantitative evolution of global research output on frailty and falls among older adults over time?
2. Which countries, institutions, and subject categories have demonstrated leadership in frailty and falls research, as evidenced by high productivity metrics?
3. Which peer-reviewed journals have published the most impactful frailty and falls research, as measured by citations, and what are their most influential articles?
4. What conceptual themes and trends can be inferred from an analysis of frequently occurring and co-occurring keywords in frailty and falls publications, and how have these evolved longitudinally?

Thus, it is believed that this bibliometric study will provide a comprehensive assessment of the state of research on frailty and falls among older adults, identifying publication trends, knowledge gaps, and promising future research directions to advance understanding in this critical area.

## Methods

**Data collection:** Relevant publications on frailty and fall were retrieved from the Scopus and Web of Science (WoS) databases on November 7^th^, 2023, using a keyword search strategy. The search terms included “frail*” AND “fall*” OR “slip and fall” based on recommendations from prior studies (25). No date restrictions were applied. All records containing cited references were exported and pre-processed using ScientoPy (version v2.1.3) software for cleaning and deduplication.

**Figure 1:**
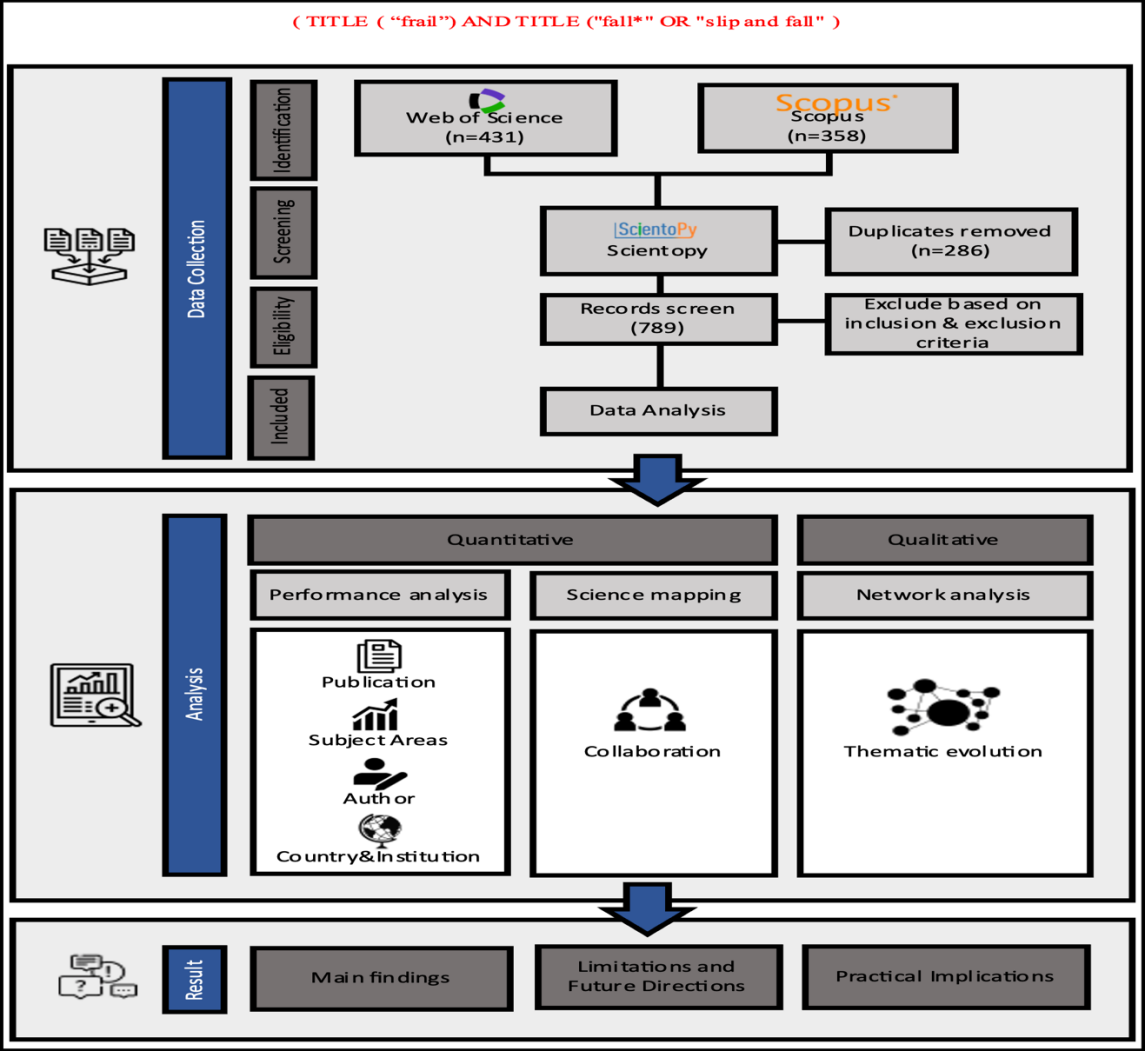
The study process diagram of the search process adapted from the previous study (27)

**Data analysis:** Figure 1 presents the study process gained and refined dataset using the bibliometric tools ScientoPy, and VOSviewer (version 1.6.19) (26). ScientoPy provided summary statistics on the loaded papers, omitted papers, duplicated papers, and final dataset composition. VOSviewer was used to visualize collaboration networks, create maps based on co-occurrence, and analyze trends in keywords. Maps were generated for country collaboration, institutional collaboration, co-authorship, keyword co-occurrence, and temporal keyword trends.

Key pre-processing results from ScientoPy indicated 789 original papers loaded, with 158 omitted and 286 duplicates removed, leaving a final dataset of 345 papers (84.6% from WoS, 15.4% from Scopus). Integrating data mining, information visualization, and contextual interpretation enabled a comprehensive bibliometric analysis to address the stated research questions on publication trends, leading contributors, influential works/journals, and conceptual themes and evolutions based on keyword analysis. Both quantitative metrics and qualitative visualizations were leveraged to synthesize a holistic overview of the frailty literature focused on older populations.

## Results

This section discusses each of the research questions and interprets the results.

*RQ1: What has been the quantitative evolution of global research output on frailty and falls among older adults over time?*

**Figure 2:**
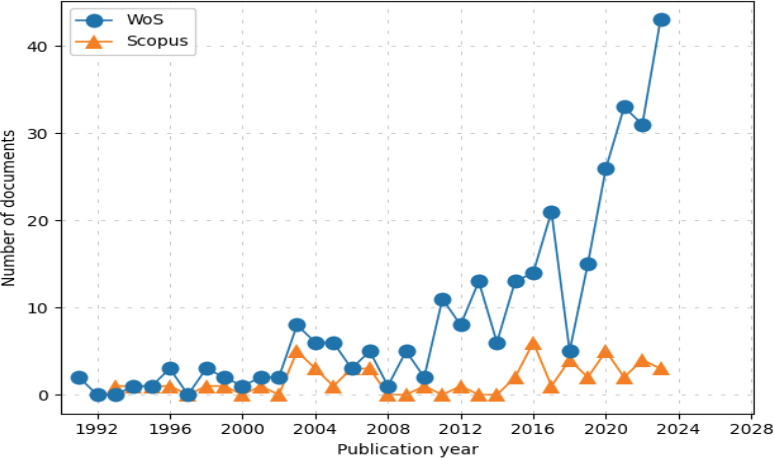
The growth of publications on frailty and fall from 1990-2023

Figure 2 depicts an analysis of the quantitative evolution of global research output on frailty and fall over time. The dataset contains publication counts from the Web of Science (WoS) and Scopus databases from 1990-2023. It provides a comprehensive overview of two prominent academic databases, Web of Science (WoS) and Scopus, from 1990 to 2023. In 1990, Web of Science had 292 records, displaying an annual growth rate (AGR) of 5%. Its h-index, a measure of impact and productivity, stood at 56, highlighting its strong influence in the academic community. Over the years, WoS experienced fluctuations in the number of records, with a peak of 43 in 2023 and a low of 0 in 1990. The AGR varied annually, reaching its highest point at 13.3% in 2021, while the h-index steadily increased, reaching 43 in 2023.

On the other hand, in 1990, Scopus contained 53 records with a modest AGR of 0.5% and a h-index of 12. Similarly, to WoS, the number of records in Scopus fluctuated, reaching a peak of 33 in 2023. The AGR experienced fluctuations, with a maximum of 13.2% in 1993, and the h-index increased, reaching 13 in 2023. In summary, both databases displayed growth in the number of records over the years, with Web of Science maintaining a higher number of records and a generally higher h-index, indicating its broader impact and recognition. Scopus, while having a lower number of records, also demonstrated growth and an increasing h-index, signifying its growing influence in the academic world. WoS has more thorough coverage of older and prestigious literature in the frailty and falls field, which would increase its credibility with scholars and researchers (28). In fact, the coverage extends back to the early 1990s, as opposed to Scopus, which began in 1993. Apart from that, WoS is an evolving record of the dynamic world of scholarly communication with new source content continually added, including citations. Web of Science's citation analysis tools, such as Journal Citation Reports (JCR), provide deep insights into the impact and influence of journals in specific academic areas (29).

*RQ2: Which countries, institutions, and subject categories have demonstrated leadership in frailty and falls research, as evidenced by high productivity metrics*

**Figure 3:**
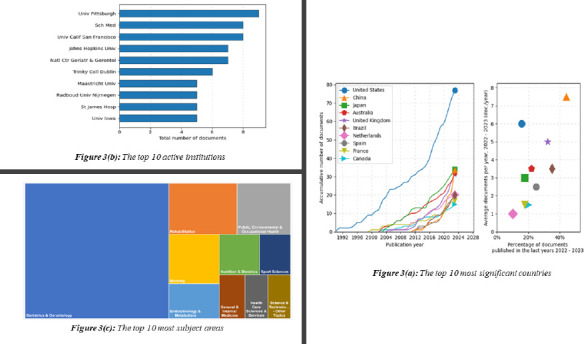
(a) countries; (b) active institutions and (c)subject areas

The bibliometric analysis reveals that frailty research has been led by several key countries, institutions, and research domains. The United States consistently had a high number of records in the database, with 77 in 1990 and 77 in 2023. Despite the high numbers, the AGR (Annual Growth Rate) is negative at -0.5% in 2023, indicating a slight decline in record numbers over the years. See Figure 3(a). The country has a moderate h-index of 33, indicating a substantial impact. The fact that the United States is a world leader in scientific research across areas of frailty research is a result of several causes: (1) significant changes in the population due to an increase in the number of older people (30), which has sparked research into related health issues such as frailty; (2) substantial financial support for frailty research from governmental, corporate, and academic institutions, made possible by a well-established infrastructure (31,32).

China, while having 34 records in 1990, has experienced significant growth over the years with a high AGR of 2.5% in 2023. The h-index for China is relatively low at 13, indicating room for further growth in research impact. An increasing number of frailty research issues among older adults in China concluded that frailty is prevalent and associated with a high incidence of fall (33-35).

Japan on the other hand is another Asian country that remained relatively stable on frailty and fall issues over the years, with 34 records in 2023. The country has maintained a moderate h-index of 12, suggesting consistent research impact. In a similar vein, given its rapidly aging population and health reforms aimed at addressing the issues of aged care, Japan's growing frailty publishing rates correspond with policy emphasis (36). Overall, it may be said that United States, China and Japan experiencing high frailty and fall research output owing to its well-established research infrastructures, large aging population and commitment to gerontology and related fields have led to a notable number of publications.

Next, Figure 3(b) presents the top 10 most active institutions in frailty and fall issues worldwide. The key institutions are located in The United States, Ireland, the Netherlands, and the United Kingdom. Pittsburgh University stands out and has published a total of 9 research papers. The Average Daily Yield (ADY) and Publications Daily (PDLY) metrics are relatively high, suggesting consistent research activity. The h-index of 7 indicates a moderate research impact. Meanwhile, Emory University School of Medicine, Atlanta, Georgia, United States has published 8 research papers. The AGR and ADY are relatively low, and PDLY is zero, indicating minimal recent growth. However, the h-index is high at 8, suggesting a significant research impact. The University of California has published 8 research papers. The AGR is negative (-1), indicating a decline in publications over time. The h-index is 7, showing a moderate research impact.

All the top three universities are situated at the United States, and all are prominent institutions with a strong commitment to frailty research as well as a strong department or researchers specializing in gerontology, aging, and related fields (37).

Concerning the subject area, geriatric and gerontology is the most dominant subject category with over 156 publications. This indicates its high relevance to frailty and fall issues. Geriatrics deals specifically with the health and care of older individuals, making it a crucial field for understanding and addressing frailty and fall-related challenges in the elderly. Rehabilitation, on the other hand, has 28 publications, a much lower number compared to geriatric and gerontology. Nevertheless, it focuses on enhancing and regaining health and function following disease or trauma, and therefore has great potential in frailty and falls research. Additionally, with 22 publications, public, environmental, and occupational health might not be as prioritized in this dataset as Geriatrics and Gerontology, based on the comparatively lower overall value. However, the topic may cover a wider range of elements that contribute to frailty and falls, such as workplace safety precautions, environmental changes, and public health initiatives to avoid falls and frailty in different populations.

The study of frailty and falls involves a multidisciplinary approach that integrates ideas from numerous disciplines including nursing, endocrine and metabolism, nutrition status, general medicine and healthcare technologies. Researching frailty and falls requires cooperation between several academic fields. The development of comprehensive techniques to evaluate, prevent, and treat frailty and falls in older populations is contingent upon the adoption of a multidisciplinary approach. This will ultimately promote healthy aging and enhance the quality of life for older persons in general.

*RQ3: Which peer-reviewed journals have published the most impactful frailty and falls research, as measured by citations, and what are their most influential articles?*

Table 1 presents the most impactful journals and most influential articles on frailty and fall research, with the most notable being The Journal of The American Geriatric Society with over 837 citations to the 1996 article by Wolf et al. on the effects of Tai Chi and computerized balance training in reducing fall and frailty older in older adults. This paper found that Tai Chi can improve biomedical and psychosocial indicators of frailty in older people and reduce the risk of multiple falls (38). The BMC Geriatric – A paper by Joosten et al., with a total of 132 citations found that frailty, as measured by the CHS index, is a significant risk factor for 6-month mortality in hospitalized older patients (39). However, frailty was not found to be a risk factor for in-hospital delirium or falls.

**Table 1:** The top 10 journals with their most influential articles

JOURNALS	CITE SCORE 2022	SJR 2022	SNIP 2022	CITED BY	MOST INFLUENTIAL ARTICLES	INSIGHT
Journal of the American Geriatrics Society	10.4	2.054	2.072	837	Reducing frailty and falls in older persons: An investigation of Tai Chi and computerized balance training (38).	The paper investigates the effects of Tai Chi and computerized balance training on reducing frailty and falls in older persons.
BMC Geriatrics	5.1	1.127	1.546	132	Prevalence of frailty and its ability to predict in hospital delirium, falls, and 6-month mortality in hospitalized older patients (39).	The paper found that frailty, as measured by the CHS index, is a significant risk factor for 6-month mortality in hospitalized older patients. However, frailty was not found to be a risk factor for in-hospital delirium or falls.
Journal of Nutrition Health & Aging	8.0	1.269	1.41	96	Frailty in relation to the risk of falls, fractures, and mortality in older Chinese adults: Results from the Beijing Longitudinal Study of Aging (40).	Frailty, as measured by a frailty index (FI), was found to be associated with an increased risk of falls, fractures, and mortality in older Chinese adults, according to the Beijing Longitudinal Study of Aging.
Archives Of Gerontology And Geriatrics	6.8	1.008	1.351	36	Efficacy of simple home-based technologies combined with a monitoring assistive center in decreasing falls in a frail elderly population (results of the Esoppe study) (41).	The Esoppe study found that the use of light path coupled with tele-assistance service significantly reduced the incidence of unintentional falling at home among frail elderly population.
Geriatric Nursing	2.9	0.666	0.836	28	Frailty as a predictor of future falls in hospitalized patients: A systematic review and meta-analysis (42).	Frailty was found to be significantly associated with future falls in hospitalized patients, according to the systematic review and meta-analysis.
Journal of Frailty & Aging	5.6	0.818	1.002	31	Frailty and fear of falling: The FISTAC Study (43).	Fear of falling syndrome is associated with female sex, frailty, depressed mood, social risk, muscle strength and power, physical function, number of drugs used, and orthostatic hypotension in older adults with a history of falls.
Journal of the American Medical Directors Association	9.6	1.794	1.97	291	Frailty as a Predictor of Future Falls Among Community-Dwelling Older People: A Systematic Review and Meta-Analysis (12).	Frailty is a significant and independent predictor of short-term future falls in community-dwelling older people, even in those who appear to be ageing well.
Topics in Geriatric Rehabilitation	1.1	0.197	0.299	33	The fear of falling syndrome: Relationship to falls, physical performance, and activities of daily living in frail older persons (44).	Fear of falling is prevalent among older men with impaired mobility and is associated with diminished physical performance, increased disability, and greater depression, but is not necessarily predictive of future falls.
Aging Clinical and Experimental Research	7.3	0.982	1.306	57	Relationship between dual-task related gait changes and intrinsic risk factors for falls among transitional frail older adults (45).	Changes in gait or attention-demanding task performance while dual tasking significantly increase the risk of falls in older adults, particularly frail ones.
Journals Of Gerontology Series A-Biological Sciences and Medical Sciences	9.9	1.703	1.522	528	Frailty and risk of falls, fracture, and mortality in older women: The study of Osteoporotic fractures (46).	Frailty is an independent predictor of increased risk of falls, fractures, and mortality in older women, regardless of age or BMI.

The Journal of Nutrition, Health and Aging–A paper published in 2012 among older Chinese adults in Beijing, found that frailty, as measured by a frailty index (FI), was found to be associated with an increased risk of falls, fractures, and mortality. This paper also highlights the complex relationship between frailty, falls, and mortality in the context of aging (40). Frailty characterized by reduced physiological reserves and increased vulnerability (4) can significantly contribute to an individual's risk of falling.

The Archives of Gerontology and Geriatric also published an impactful paper by Tchalla, et al with over 36 citations. This research investigated simple home-based technologies combined with a monitoring assistive center in decreasing falls among frail elderly. The results showed a significant reduction of unintentional falling incidence at home among frail elderly population (41).

Overall, research on frailty and falls in academic journals is driven by the urgency in addressing the health challenges associated with an aging population. The multidimensional nature of frailty and falls, coupled with their impact on individuals and societies, makes these topics rich areas for investigation and publication in a variety of academic disciplines and journals.

*RQ4: What conceptual themes and trends can be inferred from an analysis of frequently occurring and co-occurring keywords in frailty and falls publications, and how have these evolved longitudinally?*

The keyword co-occurrence map represents the relationships and associations between frailty and fall research. The map also reveals rich insights into the conceptual structure and evolution of frailty and fall research. Six broad thematic clusters emerge from the analysis:

Cluster 1 reveals a thorough focus on a range of topics about mobility, geriatrics, and general physical health. Research addressing the effects of aging on physical function, strategies to preserve or enhance balance and mobility, and the contribution of physical activity and exercise to healthy aging are probably included in this cluster.

Cluster 2 represents various aspects of geriatric health, with a particular emphasis on cardiovascular health, musculoskeletal issues, frailty, and overall quality of life. The presence of “blood pressure” in the cluster indicates a likely focus on cardiovascular health in the geriatric population. The keywords “falls,” “fracture,” and “osteoporosis” suggest a significant focus on the prevention of falls and associated fractures in the geriatric population. Studies in this cluster may explore risk factors for falls, interventions to prevent falls, and the impact of falls on bone health.

Cluster 3 suggests a focused research area within geriatrics that addresses the intersection of cognitive and physical health in older adults. The keywords “depression”, and “cognitive frailty” likely explore the complex interplay between mental health, physical function, and Overall well-being. The intersection of cognitive and physical health, along with considerations for mental well-being, nutritional status, and the older adult population's diversity, underscores the complexity of addressing frailty in geriatric research.

Cluster 4 indicated a focus on community-based interventions, rehabilitation strategies, and the use of rigorous experimental designs to assess the effectiveness of interventions for frail elderly individuals.

Cluster 5 indicates a research focus on understanding the factors influencing cognitive function, with an emphasis on identifying and addressing risk factors that may contribute to cognitive decline or impairment. The keywords “cognition” and “risk factor” imply a central focus on the mental processes involved in identifying factors that may pose a risk to cognitive function. These risk factors could include various elements such as lifestyle, health conditions, genetics, and environmental influences that may contribute to cognitive decline or impairment.

Cluster 6 proposed a study focusing on investigating the connection between mortality and disability, especially considering aging and health. The keywords “disability” and “mortality” focus on the measurement and characterization of disability levels among frail older adults and the risk of mortality.

The prominent appearance of the keyword's “fall” and “older adults” suggests that falls are a common concern in aging populations, and their association with frailty adds a layer of complexity.

## Discussion

### Implication for research

Bibliometric analysis studies have significant implications in various fields that help researchers identify research trends, and structure of research fields, highlight emerging themes, and suggest future research directions (24,47). By mapping out the authors, area of studies, institutions, and country, bibliometric studies can highlight potential collaboration and research networks. Bibliometric analysis of frailty and fall research provides several novel insights. It reveals rising publication trends, with the literature growing steadily since 1990. The analysis maps the global landscape, showing the United States, China, and Japan as dominant countries. It highlights prolific institutions such as Pittsburgh University pioneering frailty research. The study also unveils leading journals like the Journal of the American Geriatrics Society publishing high-impact work. Most interestingly, keyword analysis uncovers conceptual clusters focusing on geriatric physical health, cardiovascular health, cognition, interventions, and mortality. The temporal evolution of keywords like “frailty” and “fall” underscores their research prominence.

The findings offer tangible implications to shape frailty and fall research, clinical practice, and health policies for older adults. Quantitative growth trends justify ongoing investments to advance frailty science. The research landscape presented can aid international collaboration and identify potential partners. Knowledge of prolific publishers and impactful studies helps identify authoritative evidence sources to guide clinical protocols and health policies aimed at assessing and managing frailty. Conceptual keyword clusters pinpoint priority research domains warranting attention when planning future projects on frailty-related mobility, cardiovascular health, cognition, rehabilitation, and mortality. The findings of this bibliometric analysis have significant implications for clinical practice and policymaking related to frailty and falls among older adults. By identifying leading journals and impactful studies, clinicians and policymakers can access authoritative sources of evidence to inform clinical protocols, practice guidelines, and health policies aimed at assessing and managing frailty and falls.

Furthermore, the conceptual themes emerging from the keyword analysis can guide the development of comprehensive, multidisciplinary approaches to address the diverse aspects of frailty, including physical, cognitive, and psychosocial factors. This holistic perspective is crucial for designing effective interventions and care models that cater to the complex needs of frail older adults.

### Study's limitations and potential bias

This paper covers two databases, WoS and Scopus which may limit the extensive search of frailty and fall research. Thus, adding more databases for example Google Scholar, PubMed, and other discipline-specific databases will enhance the completeness and accuracy of this topic. In addition, it is crucial to incorporate regional databases to capture literature from non-English speaking countries or regions that are underrepresented in global databases as most of the databases favor English language publications. Another potential limitation is the inherent bias of the search strategy employed. The choice of search terms, such as “frail*” AND “fall*” OR “slip and fall,” may have excluded relevant publications that used different terminology or keywords. Additionally, the exclusion of non-English publications may have introduced a language bias, underrepresenting research from non-English-speaking countries or regions.

Furthermore, bibliometric analyses are based on quantitative metrics and may not fully capture the qualitative aspects or nuances of the research being analyzed. Citation counts and impact factors, while widely used measures of research influence, can be influenced by various factors beyond the intrinsic quality or significance of the work. Several promising future directions emerge from this bibliometric study. The research growth underscores the need for ongoing investigation into frailty mechanisms, screening, assessment, and management. Future bibliometric studies could incorporate additional datasets, enrich visualizations and qualitative analyses, and prompt regular updates of the knowledge maps as frailty research proliferates. Longitudinal analyses will further illuminate trajectories across geographies, disciplines, and focus areas.

Collaborations should be strengthened between identified leading countries and institutions to increase knowledge sharing. High-impact publication venues and studies offer a roadmap for producing meaningful frailty research. Conceptual keyword clusters suggest potentially fruitful research areas to be explored further, including multidomain frailty interventions and reducing cognitive frailty. Big data analytics, machine learning, and precision medicine approaches could provide fresh perspectives. Ultimately, advancing frailty science will require sustained, coordinated efforts between researchers, clinicians, and policymakers worldwide.

### Recommendations to address limitations

To address these limitations and potential biases, several recommendations can be considered for future bibliometric studies in this field:

1. Expand the data sources: Incorporate additional databases, such as Google Scholar, PubMed, and discipline-specific databases, to ensure broader coverage and reduce the risk of selection bias.
2. Include regional and non-English databases: To capture literature from non-English-speaking countries or regions, it is crucial to incorporate regional databases and include non-English publications in the analysis.
3. Refine the search strategy: Employ a more comprehensive and iterative search strategy, involving subject matter experts and librarians, to ensure the inclusion of relevant publications that may use different terminology or keywords.
4. Combine bibliometric analysis with qualitative methods: Integrate qualitative methods, such as expert consultations, focus groups, or content analysis, to complement the quantitative bibliometric data and provide a more nuanced understanding of the research landscape.
5. Explore alternative metrics: Consider using alternative metrics, such as altmetrics or usage data, in addition to traditional citation-based measures, to capture a more comprehensive picture of research impact and influence.
6. By addressing these limitations and implementing the recommended strategies, future bibliometric studies can provide more robust and comprehensive insights into the research on frailty and fall among older adults, ultimately contributing to the advancement of knowledge and the development of effective interventions and policies in this critical area.

## Figures and Tables

**Figure 4(a):**
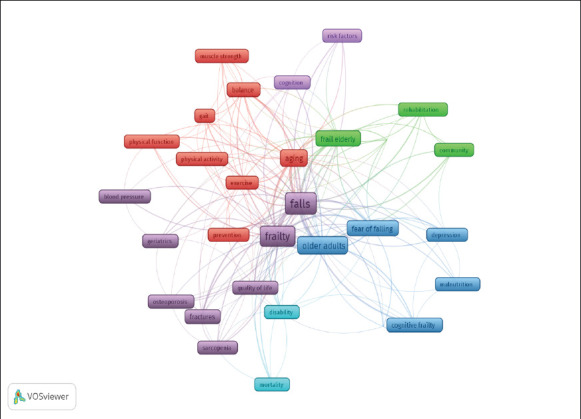
Co-occurrence Analysis of network visualizations for the authors' keywords

**Figure 4(b):**
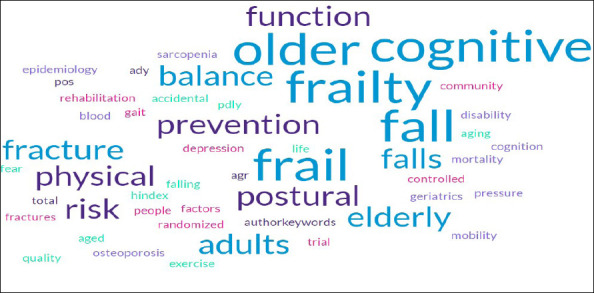
Word cloud

## References

[1] Padeiro M, Santana P, Grant M (2023). Global aging and health determinants in a changing world.

[2] Yan E, He D, Rajji TK, Chung F (2023). Cognitive impairment and its adverse outcomes in older surgical patients: an under-recognized problem!. International Anesthesiology Clinics.

[3] Brivio P, Paladini MS, Racagni G, Riva MA, Calabrese F, Molteni R (2019). From Healthy Aging to Frailty: In Search of the Underlying Mechanisms. Current Medicinal Chemistry [Internet].

[4] Church S, Rogers E, Rockwood K, Theou O (2020). A scoping review of the Clinical Frailty Scale. BMC Geriatrics.

[5] Fried L P, Tangen CM, Walston J, Newman AB, Hirsch C, Gottdiener J, Seeman T, Tracy R, Kop WK, Burke G, McBurnie MA (2001). Frailty in older adults: evidence for a phenotype. Journal of Gerontology Series A, Biological sciences, and Medical Sciences.

[6] Xiao X, Li L, Yang H, Lei P, Guo C, Cui W (2023). Analysis of the incidence of falls and related factors in elderly patients based on comprehensive geriatric assessment. Aging medicine.

[7] Chen H, Huang L, Xiang W, Liu Y, Xu JW (2023). Association between cognitive frailty and falls among older community dwellers in China: A Chinese longitudinal healthy longevity survey-based study. Frontiers in Aging Neuroscience.

[8] Morros-González E, Chacón-Valenzuela Estephanía, Vargas-Beltrán Maria Paula, Gómez Ana María, Chavarro-Carvajal Diego Andrés, Cano-Gutierrez Carlos Alberto (2023). Falls, hospitalizations, and poor self-rated health in older people with diabetes and frailty: A secondary analysis of SABE-Colombia. Research Square (Research Square).

[9] Iskandar I, Joanny A, Azizan A, Justine M (2021). The Prevalence of Sarcopenia and Its Impact on Quality of Life in Elderly Residing in the Community. Malaysian Journal of Medicine and Health Sciences.

[10] Cheng MH, Chang SF (2017). Frailty as a Risk Factor for Falls among Community Dwelling People: Evidence from a Meta-Analysis. Journal of Nursing Scholarship.

[11] Deandrea S, Lucenteforte E, Bravi F, Foschi R, La Vecchia C, Negri E (2010). Risk Factors for Falls in Community-dwelling Older People. Epidemiology.

[12] Kojima G (2015). Frailty as a Predictor of Future Falls Among Community-Dwelling Older People: A Systematic Review and Meta-Analysis. Journal of the American Medical Directors Association.

[13] Mengste Yingesu Lemma, Belete Gizachew Tilahun, Eticha Biruk Lelisa, Zeleke Tarekegn Cheklie (2023). Self-Reported Fall-Related Injury and Its Associated Factors among Adults with Visual Impairment Attending St. Paul's Hospital Millennium Medical College, Addis Ababa, Ethiopia. Ethiopian Journal of Health Sciences.

[14] Salis F, Mandas A (2023). Physical Performance and Falling Risk Are Associated with Five-Year Mortality in Older Adults: An Observational Cohort Study. Medicinalithuania.

[15] Niksolat F, Zandieh Z, Roshani F, Larijani SS, Mirfakhraee H, Bahadori F (2022). Geriatric syndromes among patients with rheumatoid arthritis: A comparison between young and elderly patients. Ethiopian Journal of Health Sciences.

[16] Murman D (2015). The Impact of Age on Cognition. Seminars in Hearing.

[17] Sugimoto T, Arai H, Sakurai T (2021). An update on cognitive frailty: Its definition, impact, associated factors and underlying mechanisms, and interventions. Geriatrics & Gerontology International.

[18] Azizan Azliyana, Anum Asilah, Faisal Ameera, Karnadipa Triana (2023). The Effects of Exercise and Reminiscence Therapy on Depression and Quality of Life Among the Older Adults with Mild Alzheimer's Disease. Journal of Advanced Research in Applied Sciences and Engineering Technology.

[19] Jungen P, Batista JP, Kirchner M, Habel U, Bollheimer L Cornelius, Huppertz C (2022). Variability and symmetry of gait kinematics under dual-task performance of older patients with depression. Aging Clinical and Experimental Research.

[20] Byun M, Kim J, Kim JE (2021). Physical and Psychological Factors Contributing to Incidental Falls in Older Adults Who Perceive Themselves as Unhealthy: A Cross-Sectional Study. International Journal of Environmental Research and Public Health.

[21] de Souza LF, Canever JB, Moreira B de S, Danielewicz AL, de Avelar NCP (2022). Association Between Fear of Falling and Frailty in Community-Dwelling Older Adults: A Systematic Review. Clinical Interventions in Aging.

[22] Yang Z, Lin H, Jiang G, Chu YH, Gao J, Tong Z (2023). Frailty Is a Risk Factor for Falls in the Older Adults: A Systematic Review and Meta-Analysis. Journal of Nutrition Health & Aging.

[23] Dagli Namrata, Patel B, Dagli Rushabh, Adnan N, Ahmad R, Haque M (2023). Bibliometric analysis and visualization of research on nanotechnology in dentistry from 1999 to 2022. Journal of applied pharmaceutical science.

[24] Jena JR, Panigrahi RR, Shrivastava AK (2023). A bibliometric analysis on financial engineering studies. International Journal of Financial Engineering.

[25] Li J, Goerlandt F, Li KW (2019). Slip and Fall Incidents at Work: A Visual Analytics Analysis of the Research Domain. International Journal of Environmental Research and Public Health.

[26] Aria M, Cuccurullo C (2017). bibliometrix: An R-tool for comprehensive science mapping analysis. Journal of Informetrics.

[27] Azizan Azliyana, Abdullah Khairul Hafezad, Rahayu Sri, Rusli N, Azizah Nurona, Tarmidzi Nornajehah (2023). Reshaping Healthcare: A Bibliometric Analysis of Lessons Learned in Post-COVID-19 Health Policy. Kesmas: Jurnal Kesehatan Masyarakat Nasional.

[28] Joshi A (2016). COMPARISON BETWEEN SCOPUS & ISI WEB OF SCIENCE. Journal Global Values.

[29] Solari A, Magri MH (2000). A New Approach to the SCI Journal Citation Reports, a System for Evaluating Scientific Journals. Scientometrics.

[30] Vespa J, Medina L, Armstrong D (2020). Demographic Turning Points for the United States: Population Projections for 2020 to 2060 Population Estimates and Projections Current Population Reports [Internet].

[31] Walston J, Robinson TN, Zieman S, McFarland F, Carpenter CR, Althoff KN (2017). Integrating Frailty Research into the Medical Specialties-Report from a U13 Conference. Journal of the American Geriatrics Society.

[32] Lanahan L, Graddy-Reed A, Feldman MP (2016). The Domino Effects of Federal Research Funding. PLOS ONE.

[33] Cai Y, Xu W, Xiao H, Liu H, Chen T (2020). Correlation between Frailty and Adverse Outcomes Among Older Community-Dwelling Chinese Adults: The China Health and Retirement Longitudinal Study. The journal of nutrition, health & aging.

[34] Huang Y, Guo X, Du J, Liu Y (2021). Associations Between Intellectual and Social Activities With Frailty Among Community-Dwelling Older Adults in China: A Prospective Cohort Study. Frontiers in Medicine.

[35] Zeng X, Meng L, Jia N, Shi J, Zhang C, Li Y (2023). Epidemiological status and associated factors of frailty and pre-frailty in older adults with asthma in China: A national cross-sectional study. Frontiers in Public Health.

[36] Hasegawa T, Matsumoto K, Onishi R, Hirata K (2020). Social and health sector reform towards 2040 in Japan. Public Administration and Policy.

[37] Rahayu Umi Budi, Rahman F, Setiyadi Noor Alis, Azizan Azliyana (2021). Exercise and Physical Health in Survivors of COVID-19: A scoping review. Journal of medicinal and chemical sciences.

[38] Wolf SL, Barnhart HX, Kutner NG, McNeely E, Coogler C, Xu T (1996). Reducing Frailty and Falls in Older Persons: An Investigation of Tai Chi and Computerized Balance Training. Journal of the American Geriatrics Society.

[39] Joosten E, Demuynck M, Detroyer E, Milisen K (2014). Prevalence of frailty and its ability to predict in hospital delirium, falls, and 6-month mortality in hospitalized older patients. BMC Geriatrics.

[40] Fang X, Shi J, Song X, Mitnitski A, Tang Z, Wang C (2012). And mortality in older Chinese adults: Results from the Beijing longitudinal study of aging. The journal of nutrition, health & aging.

[41] Tchalla AE, Lachal F, Cardinaud N, Saulnier I, Bhalla D, Roquejoffre A (2012). Efficacy of simple home-based technologies combined with a monitoring assistive center in decreasing falls in a frail elderly population (results of the Esoppe study). Archives of Gerontology and Geriatrics.

[42] Lan X, Li H, Wang Z, Chen Y (2020). Frailty as a predictor of future falls in hospitalized patients: A systematic review and meta-analysis. Geriatric nursing.

[43] Esbrí Víctor M, Huedo Rodenas I, López Utiel M, Navarro López J, Martínez Reig M, Serra Rexach J, Romero Rizos L, Abizanda P (2017). Frailty and Fear of Falling: The FISTAC Study. The Journal of frailty & aging.

[44] Chandler J, Duncan P, Sanders L, Studenski S (1996). The fear of falling syndrome: Relationship to falls, physical performance, and activities of daily living in frail older persons. Topics in Geriatric Rehabilitation.

[45] Beauchet O, Dubost V, Herrmann F, Rabilloud M, Gonthier R, Kressig RW (2005). Relationship between dual-task related gait changes and intrinsic risk factors for falls among transitional frail older adults. Aging Clinical and Experimental Research.

[46] Ensrud KE, Ewing SK, Taylor BC, Fink HA, Stone KL, Cauley JA, Tracy JK, Hochberg MC, Rodondi N, Cawthon PM (2007). Frailty and Risk of Falls, Fracture, and Mortality in Older Women: The Study of Osteoporotic Fractures. The Journals of Gerontology Series A: Biological Sciences and Medical Sciences.

[47] Azizan Azliyana (2023). Mapping the Muscle Mass: A Birds-Eye View of Sarcopenia Research Through Bibliometric Network Analysis. International journal of disabilities sports & health sciences.

